# Linear synngocystadenoma papilliferum of the limb: a rare localization of an uncommon tumour^☆^

**DOI:** 10.1016/j.abd.2021.09.024

**Published:** 2023-06-29

**Authors:** Ming Yao, Lang Rao

**Affiliations:** Department of Dermatology, Chengdu Second People's Hospital, Chengdu, Sichuan Province, China

Dear Editor,

Syringocystadenoma Papilliferum (SCAP) is a benign adnexal neoplasm that most frequently arises from an organoid nevus on the head and neck. It usually occurs during childhood or adolescence, varying in morphological character from smooth and flat to verrucous form. Most reported cases in the literature are single lesions presenting as a solitary raised warty plaque, and less commonly multiple papules. Here we report a case of multiple SCAP with a warty surface presenting on the limb distributed along a Blaschko line and without pre-existing lesions in an adult.

A 45-year-old female presented with several pink nodules on the left upper limb for seven years. The lesions were pruriginous and prone to bleed after scratching. Physical examination revealed multiple, verrucous papules, measuring 1 to 2.5 cm in the left upper extremity following a line of Blaschko. Central umbilication was seen in several lesions (Fig. 1A, B). She was misdiagnosed at another hospital with verruca vulgaris, laser was used to remove some of the lesions but soon recurred. One of the lesions was surgically excised and histopathology was performed. Features of SCAP were identified, with the tumor located in the superficial layer of the dermis without connection to the overlying epidermis, composed of cystadenoma-like structures and folded papillary structures. The cystic spaces and papillary structures were lined with single columnar epithelium and surrounded by a layer of small cuboidal myoepithelial cells, forming a special double-layer structure (Fig. 1C, D). DNA tested for Human Papillomavirus (HPV) was negative. After excision, there was no recurrence or new lesions at the 3-month and 6-month follow-ups. As the patient did not want to excise the other papules, we arranged for a subsequent visit after 6 months.

**Figure 1:**
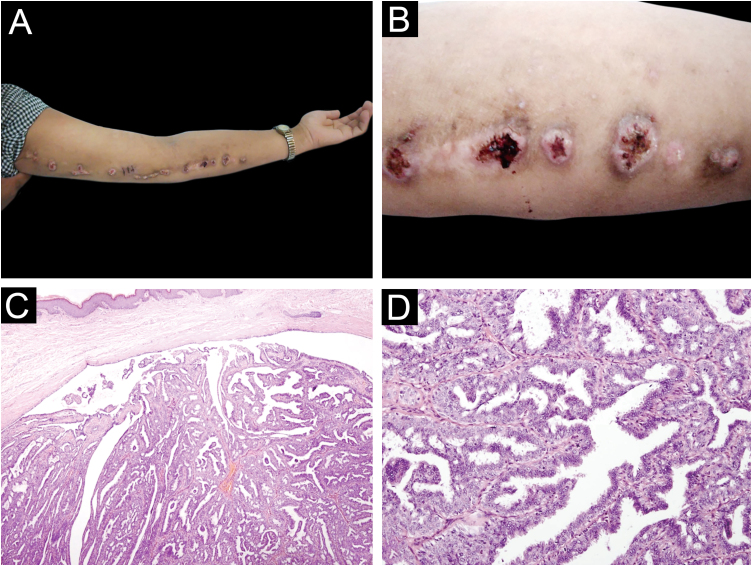
(A, B) Several normochromic papules, some with a warty surface, with a diameter of about 1 ∼ 2.5 cm on the left upper limb, arranged in a linear pattern. (C) The tumor is located in the superficial dermis without connection to the overlying epidermis, composed of cystadenoma-like structures and folded papillary structures. (Hematoxylin & eosin, ×40). (D) The cystic spaces and papillary structures are lined with single columnar epithelium and surrounded by a layer of small cuboidal myoepithelial cells, forming a special double-layer structure. (Hematoxylin & eosin, ×400)

SCAP was first described by Stokes in 1917. The pathogenesis of SCAP remains unclear, HPV DNA and mutations in the RAS/mitogen-activated protein kinase signaling pathway have been detected.^1^, ^2^ In our case we failed to identify HPV infection though the lesions show verrucous growths. SCAP frequently arises in puberty within organoid nevi in the head and neck region. As far as we have observed, there have been 17 previous cases of linear SCAP reported in the literature in English, and only two of these cases developed in adults, aged 21 and 34 respectively. The reported 17 cases include 10 females and 7 males, 6 cases occurred in the head and neck, 5 cases were in the trunk, 5 cases were in the extremities and one case was in the inguinal fold. In the extremities, 3 were found on the leg and 2 on the upper limb.^3^, ^4^, ^5^ The unique features of our case are Blaschkolinear distribution, the localization on the upper limb, late-onset in an adult, and the tumor without connection to the overlying epidermis. So far, neither organoid naevus nor epidermal naevus has been demonstrated in the linear form of SCAP. Therefore, multiple linear SCAP may represent a distinct clinical form, the relationship of linear SCAP with organoid naevus or other adnexal tumors needs further investigation.

## Financial support

None declared.

## Author's contributions

Ming Yao: Pathological analysis, article writing and figure editing; literature search; approval of the final version of the manuscript; data collection, analysis and interpretation; critical review of the manuscript.

Lang Rao: Pathological analysis, article writing and figure editing; concept determination; approval of the final version of the manuscript; data collection, analysis and interpretation; critical review of the manuscript.

## Conflicts of interest

None declared.
